# Resting-state functional MRI study of conventional MRI-negative intractable epilepsy in children

**DOI:** 10.3389/fnhum.2024.1337294

**Published:** 2024-03-06

**Authors:** Xuhong Li, Heng Liu, Tijiang Zhang

**Affiliations:** Department of Radiology, Medical Imaging Center, Affiliated Hospital of Zunyi Medical University, Zunyi, China

**Keywords:** epilepsy, intractable, functional magnetic resonance imaging, resting-state, functional connectivity strength

## Abstract

**Objective:**

The study aimed at investigating functional connectivity strength (FCS) changes in children with MRI-negative intractable epilepsy (ITE) and evaluating correlations between aberrant FCS and both disease duration and intelligence quotient (IQ).

**Methods:**

Fifteen children with ITE, 24 children with non-intractable epilepsy (nITE) and 25 matched healthy controls (HCs) were subjected to rs-fMRI. IQ was evaluated by neuropsychological assessment. Voxelwise analysis of covariance was conducted in the whole brain, and then pairwise comparisons were made across three groups using Bonferroni corrections.

**Results:**

FCS was significantly different among three groups. Relative to HCs, ITE patients exhibited decreased FCS in right temporal pole of the superior temporal gyrus, middle temporal gyrus, bilateral precuneus, etc and increased FCS values in left triangular part of the inferior frontal gyrus, parahippocampal gyrus, supplementary motor area, caudate and right calcarine fissure and surrounding cortex and midbrain. The nITE patients presented decreased FCS in right orbital superior frontal gyrus, precuneus etc and increased FCS in bilateral fusiform gyri, parahippocampal gyri, etc. In comparison to nITE patients, the ITE patients presented decreased FCS in right medial superior frontal gyrus and left inferior temporal gyrus and increased FCS in right middle temporal gyrus, inferior temporal gyrus and calcarine fissure and surrounding cortex. Correlation analysis indicated that FCS in left caudate demonstrated correlation with verbal IQ (VIQ) and disease duration.

**Conclusion:**

ITE patients demonstrated changed FCS values in the temporal and prefrontal cortices relative to nITE patients, which may be related to drug resistance in epilepsy. FCS in the left caudate nucleus associated with VIQ, suggesting the caudate may become a key target for improving cognitive impairment and seizures in children with ITE.

## 1 Introduction

Epilepsy is considered to be a serious public health problem, affecting 1–2% people worldwide (Falco-Walter, 2020). Epilepsy affects 0.5–1% of children and is a common neurological disease among children (Aaberg et al., 2017). Although significant advancements have been achieved in epilepsy treatment, approximately one third of the epilepsy patients evolve to intractable epilepsy (ITE) (Anyanwu and Motamedi, 2018). ITE, also known as medically refractory epilepsy or drug-resistant epilepsy, occurred when two appropriate and tolerated antiepileptic drugs could not control seizure (Kwan et al., 2010).

Recurrent seizures and long-term use of antiepileptic drugs cause physical and psychological damage to patients, affecting quality of life seriously. Meanwhile, it brings great economic burden to families. Sudden death from epilepsy is the main cause of death in young people with ITE (Dlouhy et al., 2016). Therefore, paying attention to and studying the abnormal brain function of ITE is important for clinical diagnosis, treatment and the study of pathological and physiological mechanisms. Typically, drug resistance in epilepsy has been investigated at the cellular or genetic levels. Nevertheless, these approaches have failed to adequately account for the underlying neurobiological mechanisms of drug resistance (Tang et al., 2017).

Lately, resting-state fMRI (rs-fMRI) has been ubiquitously adopted to study brain function (Songjiang et al., 2021; Vannest et al., 2021). Rs-fMRI is an effective approach to study entire brain functional networks, whereby statistical analysis of the blood-oxygen level dependent signal establishes degree of connectivity between regions. Additionally, there are lots of data handling methods, comprising regions of interest (ROIs) (Pressl et al., 2019; Wang et al., 2019), independent component analysis (ICA) (Boerwinkle et al., 2017; Jiang et al., 2018), regional homogeneity (ReHo) (Zhu et al., 2021; Yang et al., 2022), and amplitude of low frequency fluctuation (ALFF) (Li et al., 2017; Zhu et al., 2021). Neuroimaging research indicates that functional connections among brain regions of ITE patients are abnormal, and these abnormalities in these regions are linked to cognitive impairment (Ibrahim et al., 2014; Jiang et al., 2018). Wang et al. (2019) found that hippocampal functional connectivity levels in drug-resistant idiopathic generalized tonic-clonic seizure patients were impaired, and the degree of impairment associated with disease duration by ROI approach. The hippocampal functional connectivity levels in drug-sensitive patients showed overall compensatory enhancement. Jiang et al. (2018) found that epilepsy patients had inhibited dorsal attention network and ventral attention network functions. Based on the ICA method and the ROI method, Kay et al. (2013) speculated that default mode network (DMN) connectivity might be a biomarker of treatment resistance.

However, these methods rely on pre-selected seed regions, causing bias in research results. Moreover, prior methods analyzed only whether there was functional connectivity among brain regions; while these methods could not reflect the synchronization of neuronal activity signals in multiple brain regions at the same time and failed to calculate the total quantity of functional connections per voxel.

Therefore, we employ an updated approach to avoid these limitations. Which is called functional connectivity strength (FCS), a data-driven approach based on the voxel level to quickly calculate whole-brain functional connections. In contrast to other approaches, FCS can measure the total functional connections of each voxel; therefore, specific brain regions do not need to be preselected as seeds. FCS largely avoids artificial factors and makes up for the shortcomings of function connection methods based on ROIs (Wang et al., 2015, 2017). The FCS value reflects the functional connectivity capabilities between brain voxels. Voxels with higher FCS values have more functional connections with each other, indicating greater capacity for information transfer.

This method has been applied in studies of mild cognitive impairment (Li et al., 2016), depression (Shi et al., 2020), informant-reported subjective cognitive decline (Dong et al., 2018), schizophrenia (Wang et al., 2017), chronic subcortical stroke (Diao et al., 2020), and other neuropsychiatric diseases. A recent study showed that the abnormal FCS in major depression patients was found in DMN and cortico-limbic networks, which can offer beneficial and supplementary evidence in the exploration of pathophysiological mechanisms and treatment in depression (Shi et al., 2020). Diao discovered that FCS changes was connection-distance dependent within stroke patients. FCS in left sensorimotor cortex demonstrated positive correlation with accuracy of the Flanker test, suggesting compensatory changes of attention and executive dysfunction in these patients (Diao et al., 2020). However, there have been very few studies on epilepsy using FCS methods, and using FCS methods to study children with ITE has not yet been reported in the literature. Therefore, we conducted this study to research the brain function of children with MRI-negative ITE adopting FCS analysis and evaluate the correlations between aberrant FCS and disease duration, and between aberrant FCS and intelligence quotient (IQ). This could provide valuable new imaging evidence to understand the pathophysiological mechanism of ITE.

## 2 Materials and methods

### 2.1 Subjects

Fifteen children with ITE and 24 children with non-intractable epilepsy (nITE) were prospectively and consecutively enrolled from the pediatric outpatient department of the Affiliated Hospital of Zunyi Medical University. Healthy controls (HCs) were enrolled, matched by age, gender, and education. The criteria for including epilepsy patients: (1) children with epilepsy aged 6–16 years diagnosed by pediatricians above the deputy director based on the 2010 International League Against Epilepsy consensus. Our patient data was collected in accordance with the 2010 International League Against Epilepsy. During epileptic seizures, 12 of the 15 patients with ITE experienced consciousness disorders, 8 had convulsions, and 3 had twitching of both hands. In the 24 nITE patients, 17 presented with consciousness disorders, 8 had convulsions of the limbs, and 6 had ankylosis of the extremities ankylosis of the extremities during seizures; (2) routine brain MRI was negative. All children had 3D-T_1_WI and FLAIR scans that passed quality control review. Images without anatomical abnormalities were assessed by 2 neuroimaging experts whose positions were above the deputy director. The criteria for exclusion were shown as follows: (1) history of substance abuse; (2) history of neuropsychiatric illnesses or head trauma; (3) fell asleep during the MRI scan; and (4) head movement over 2 mm or head rotation over 2°. All participants were right-handed. The Ethics Committee of the Affiliated Hospital of Zunyi Medical University approved the study.

### 2.2 MRI data acquisition

All subjects were examined with a 3.0T MRI scanner (GE Healthcare, Milwaukee, WI). In the process of the scans, subjects were told to rest, close their eyes, keep awake, remain still, and not to consider anything particularly. Rs-fMRI parameters: repetition time (TR) = 2,000 ms, echo time (TE) = 20 ms, flip angle (FA) = 90°, field of view (FOV) = 240 × 240 mm, matrix = 64 × 64, slice thickness = 4 mm, slice interval = 0, and slices = 33. Other sequences and parameters included fluid-attenuated inversion recovery [TR = 7,826 ms, TE = 165 ms, inversion time (TI) = 2,100 ms, FOV = 240 × 40 mm, matrix = 288 × 224, FA = 90°, and slice thickness/interval = 5.0/1.5 mm] and 3D-T_1_WI (TR = 7.8 ms, TE = 3.0 ms, TI = 450 ms, FA = 15°, FOV = 256 × 256 mm, matrix = 256 × 256 mm, and slice thickness = 1 mm).

### 2.3 Neuropsychological assessment

A neuropsychologist conducted an evaluation with the help of Chinese Wechsler Intelligence Scale for Children on the same day of the MRI scan, without knowing the MRI results. The evaluation measured the verbal, performance and full-scale intelligence quotient (IQ), generally used in China (Yu et al., 2012; Hu et al., 2023). This scale is suitable for children aged 6–16 years old. IQ scores from 110 to 119 exceed the normal range. Scores between 90 and 109 are considered within the normal range. Scores between 80 and 89 are classified as below normal, while scores between 70 and 79 are considered to be on the boundary. Scores below 69 are categorized as indicating an intellectual disability (Wechsler, 1949). Our intelligence assessment results showed that 10 children with ITE had worse auditory-verbal short term memory, word comprehension, and practical knowledge mastery compared to normal peers of the same age. Eight children with ITE showed a decrease in performance in a space graphical analysis, imaginations, hand-eye coordination, attentive executive functions and memory relative to normal peers. Relative to normal peers, 9 children with nITE had poorer auditory-verbal short term memory, word comprehension, and practical knowledge mastery. Ten children with nITE showed a decline in a space graphical analysis, imaginations, hand-eye coordination, attentive executive functions, and memory compared to their normal peers.

### 2.4 Data preprocessing

All rs-fMRI data were processed using Data Processing & Analysis for Brain Imaging (DPABI) (http://rfmri.org/dpabi) with MATLAB R2014a (https://www.mathworks.com/). The first 10 time points were removed to reach steady-state magnetization and allow each subject to adjust to the scanner noise. Then, the rest 200 time points were corrected for slice timing, head motion, and spatial normalization to the standard Montreal Neurological Institute (MNI) template (voxel size 3 × 3 × 3 mm^3^). In addition, the normalized images were spatially smoothed using a full-width at half-maximum Gaussian kernel of 8 mm. FMRI data was detrended and bandpass filtered to reduce drift and noise (0.01–0.08 Hz). Six head motion parameters and global, white matter, and cerebrospinal fluid signals were removed from the data.

### 2.5 Whole-brain functional connectivity strength

The calculation of the FCS value was performed using DPABI. The entire brain functional connectivity matrices for every subject were created by calculating Pearson's correlations between time series of all pairs of voxels. This procedure was limited to a gray matter mask created by setting a threshold of 0.2 on the average map of all GM maps from all subjects. To ensure data distribution normality, individual correlation matrices were converted to *z* score matrices by Fisher's r-to-z transformation. FCS values were calculated by summing the connections between a voxel and other gray matter voxels in the entire brain. To eliminate weak correlations due to noise, only connections with correlation coefficients at *r* > 0.2 were included in the calculation. All FCS maps were spatially smoothed using an full width Gaussian kernel of 8 mm.

### 2.6 Statistical analysis

Based on the SPSS 18.0 software, one-way analysis of variance (ANOVA) was applied to assess differences in age and education level, and sex was compared by a chi-square test among the ITE group, nITE group and control group. Statistical significance was established at *p* < 0.05. All data were tested with Shapiro-Wilk normality tests. If the data satisfied a normal distribution and homogeneity of variance, ANOVA was used; if not, the Kruskal–Wallis *H*-test was used.

Analysis of covariance (ANCOVA) was performed in a voxelwise manner across the entire brain to analyze the main effect of groups on FCS using DPABI, age, sex and education level as covariates, and then pairwise comparisons were made among the three groups. The differences in FCS values among the three groups were compared by using a threshold at *p* < 0.05 with Bonferroni corrections. Correlations between FCS values in the abnormal brain areas of the ITE group and IQ was analyzed and evaluated using a Spearman correlation analysis. Pearson correlation analysis was used to evaluate correlations between FCS in abnormal brain regions of ITE group and disease duration. And we performed Pearson correlation analysis to assess correlations between aberrant FCS of the nITE group and IQ, and between aberrant FCS and disease duration. Statistical significance was established at *p* < 0.05.

## 3 Results

The ITE group, nITE group and HC group had no prominent diversities in age, gender or education (*p* > 0.05; Table 1). ANOVA of resting-state FCS was significantly different among three groups (*p* < 0.05, Bonferroni correction).

**Table 1:** Demographic and clinical characteristics of participants.

**Variables**	**ITE ±SD (*N* = 15)**	**nITE ±SD (*N* = 24)**	**HCs (*N* = 25)**	***p*-value**
Age (years)	(11.60 ± 2.44)	(10.58 ± 2.52)	(11.80 ± 2.45)	0.21
Sex, male/female	9/6	14/10	15/10	0.99
Education years	(6.20 ± 2.27)	(5.50 ± 2.39)	(6.92 ± 2.43)	0.12
VIQ scores	(75 ± 19.73)	(93.62 ± 22.63)		0.02
PIQ scores	(72.86 ± 19.81)	(91.14 ± 13.98)		0.003
FIQ scores	(71.43 ± 21.11)	(91.95 ± 17.94)		0.004
Epilepsy duration (months)	(67.93 ± 31.99)	(37.42 ± 29.05)		
Right handedness	15	24	25	

ITE, children with intractable epilepsy; nITE, children with non-intractable epilepsy; HCs, healthy controls; SD, standard deviation; VIQ, verbal intelligence quotient; PIQ, performance intelligence quotient; FIQ, full-scale intelligence quotient.

**Figure 1:**
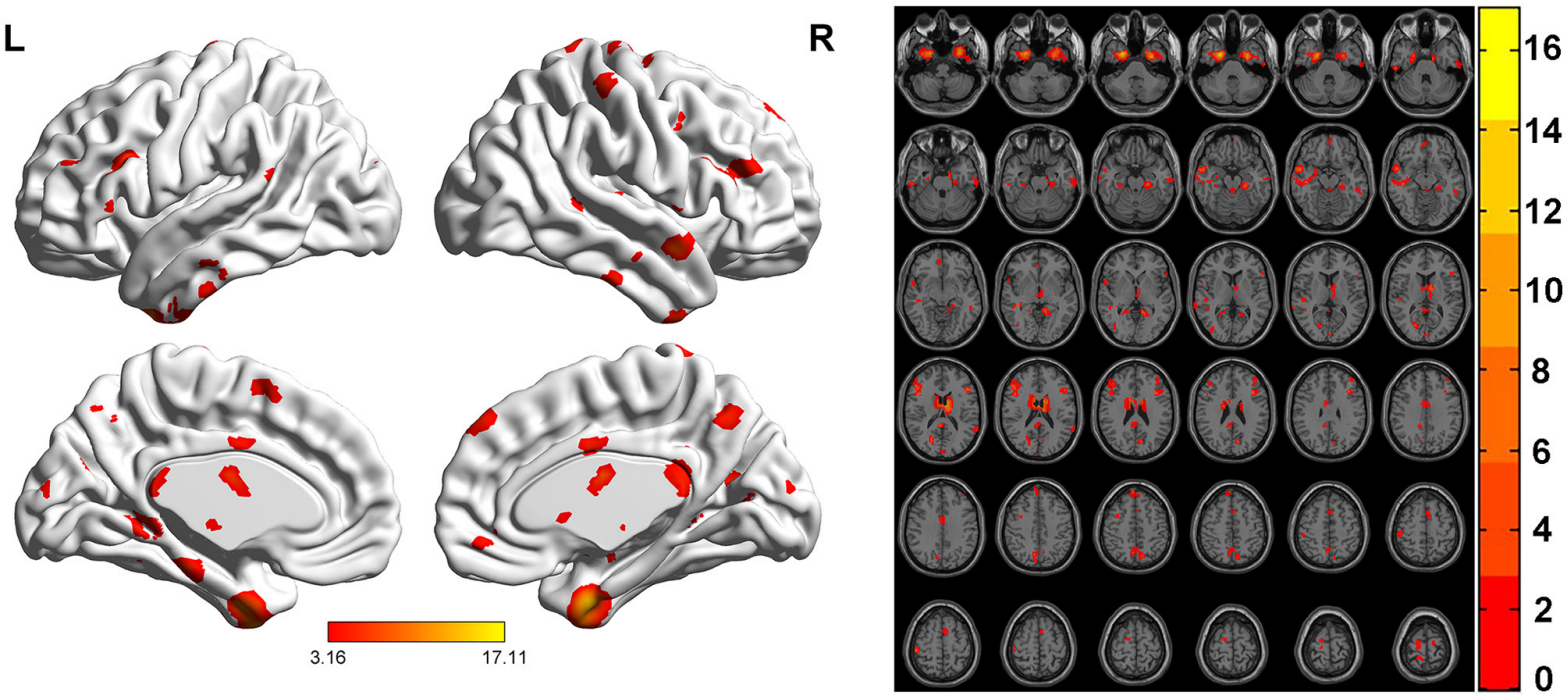
ANOVA maps of FCS values showing significant differences among the ITE group, the non-intractable epilepsy group and the control group (*p* < 0.05). The color bar indicates *F*-values.

**Figure 2:**
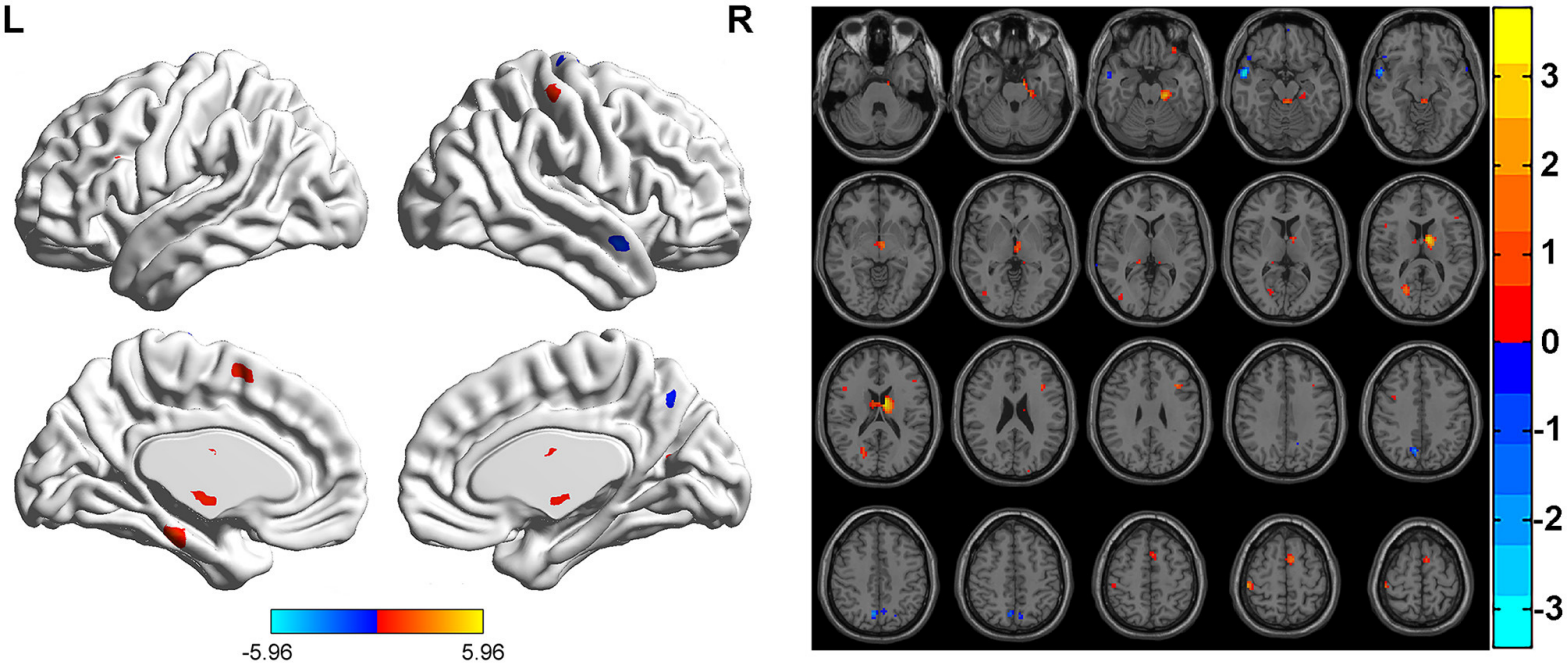
Compared with the HC group, the ITE group showed decreased FCS values in the right precentral gyrus, right temporal pole of the superior temporal gyrus, right middle temporal gyrus, right temporal pole of middle temporal gyrus, and bilateral precuneus and increased FCS values in the left triangular part of the inferior frontal gyrus, left parahippocampal gyrus, left caudate nucleus, left supplementary motor area, right postcentral gyrus, right calcarine fissure, and surrounding cortex and midbrain. The color bar indicates *t*-values.

**Figure 3:**
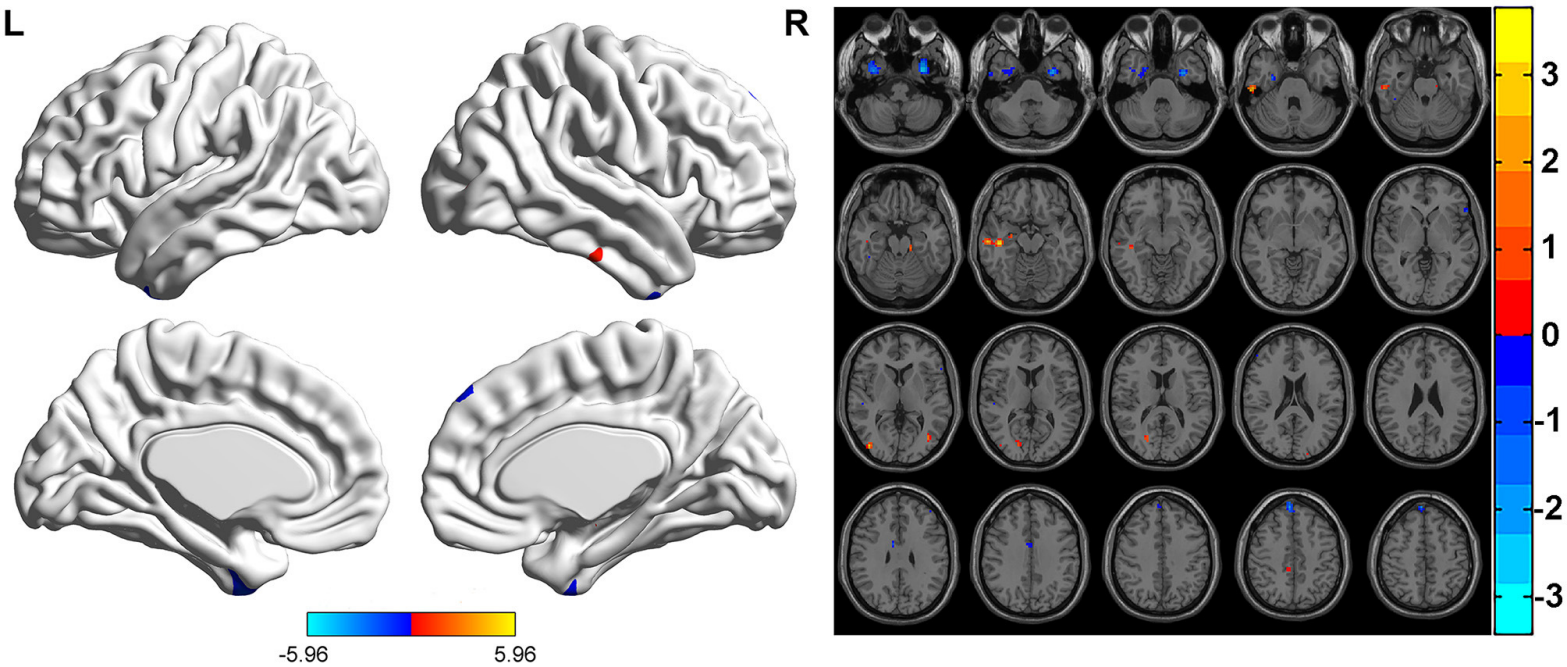
Compared with the nITE group, the ITE group displayed decreased FCS values in the right medial superior frontal gyrus, left inferior temporal gyrus and bilateral fusiform gyri and increased FCS values in the right middle temporal gyrus, right inferior temporal gyrus and right calcarine fissure and surrounding cortex. The color bar indicates *t*-values.

**Figure 4:**
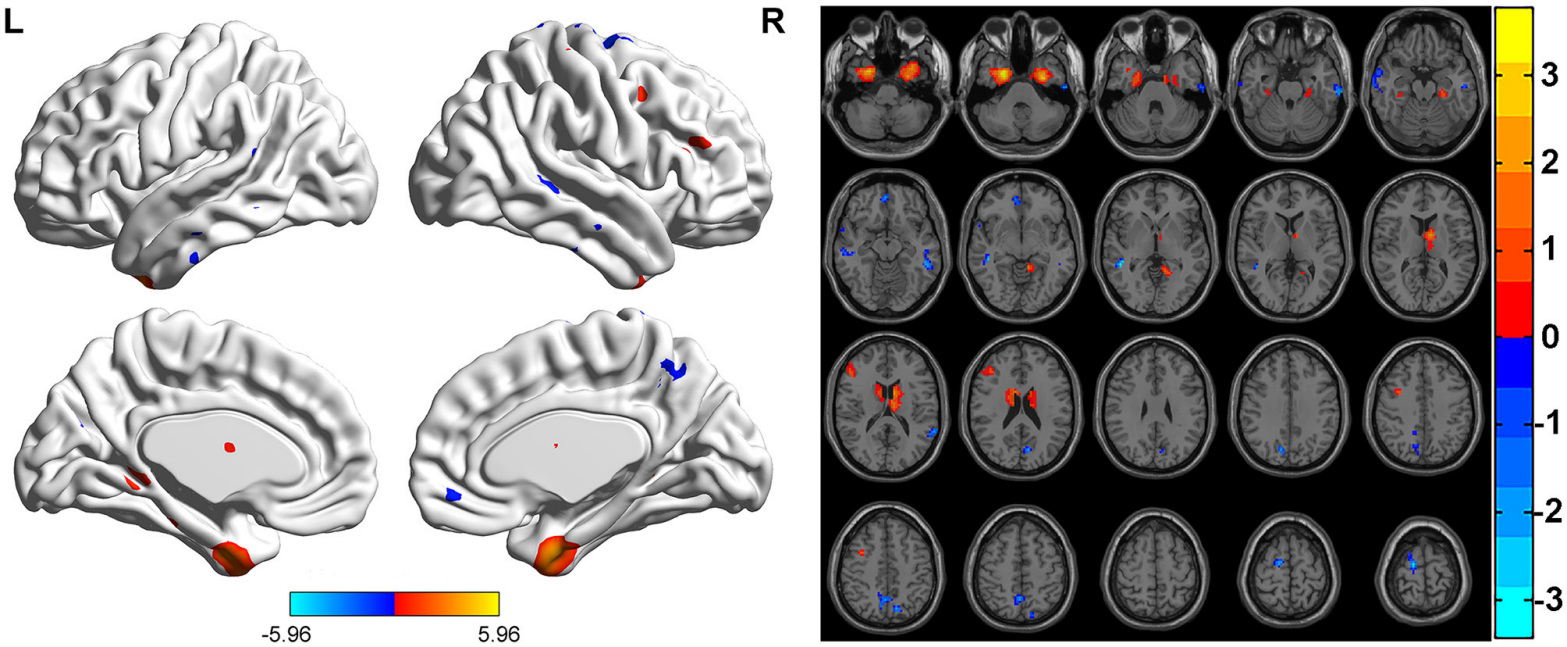
Compared with the HC group, the nITE group showed decreased FCS values in the bilateral middle temporal gyri, right orbital superior frontal gyrus, right supplementary motor area, right superior frontal gyrus and right precuneus and increased FCS values in the bilateral fusiform gyri, bilateral parahippocampal gyri, bilateral caudate nuclei, left temporal pole of the middle temporal gyrus, left thalamus, right triangular part of the inferior frontal gyrus and right middle frontal gyrus. The color bar indicates *t*-values.

**Figure 5:**
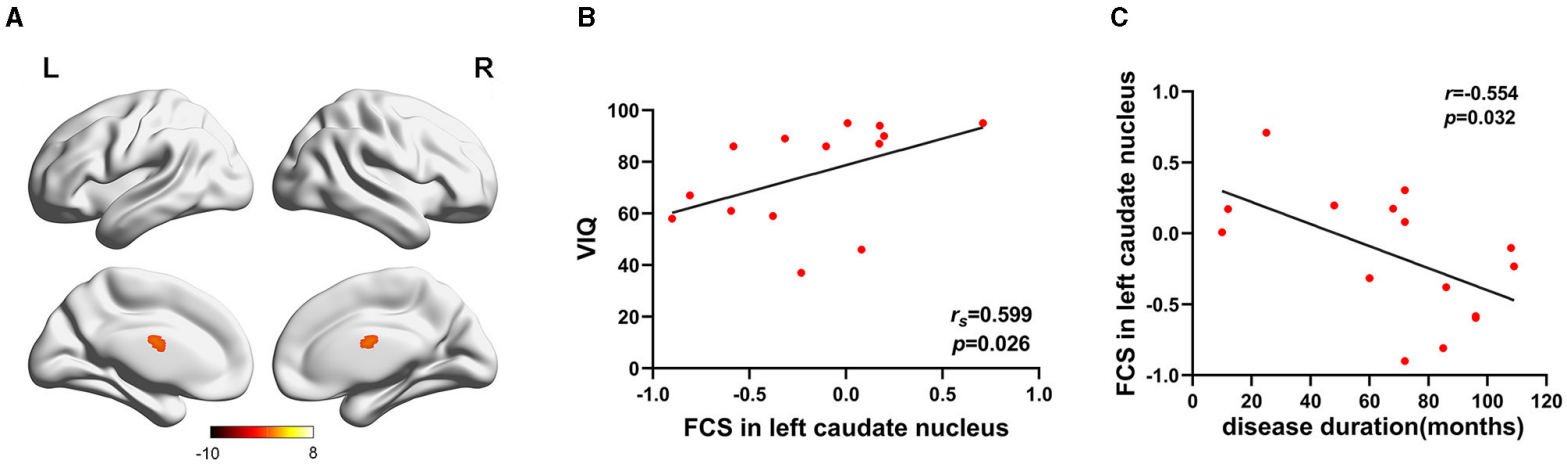
**(A)** Left caudate nucleus. The color bar indicates *t*-values. **(B)** FCS in the left caudate nucleus was positively correlated with the verbal intelligence quotient. Spearman's partial correlations were significant at *p* < 0.05. **(C)** Pearson correlation showed that FCS in the left caudate nucleus negatively correlated with disease duration.

**Figure 6:**
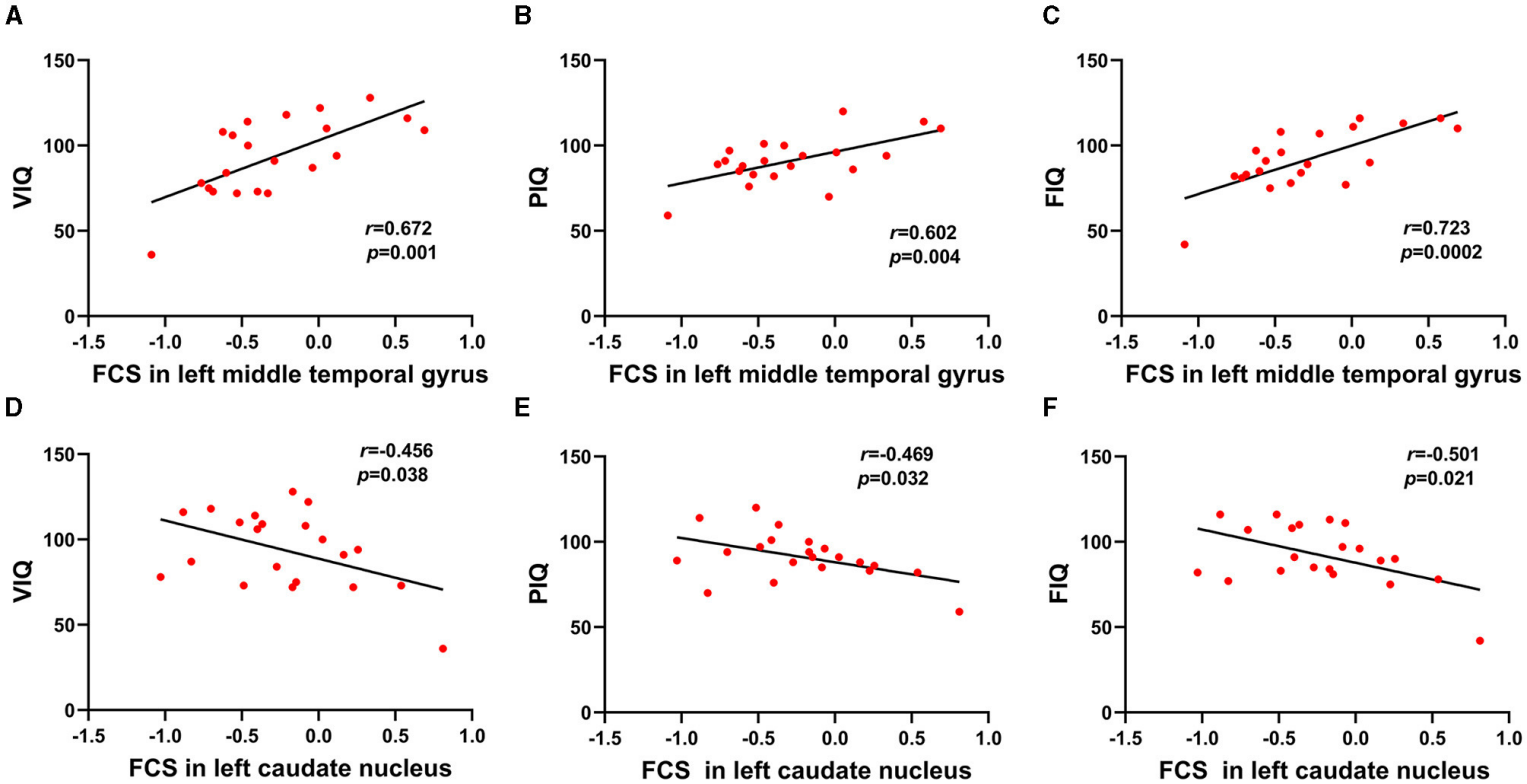
**(A–C)** FCS values in left middle temporal gyrus of the nITE group demonstrated positive correlation with VIQ, PIQ, and FIQ. **(D–F)** FCS values in left caudate nucleus of the nITE group were negatively correlated with VIQ, PIQ, and FIQ.

FCS was significantly different among three groups. Brain regions with significant differences included right superior frontal gyrus, medial superior frontal gyrus, orbital superior frontal gyrus, triangular inferior frontal gyrus, middle frontal gyrus, temporal pole of superior temporal gyrus, precentral gyrus, postcentral gyrus, middle occipital gyrus, calcarine fissure, left triangular inferior frontal gyrus, thalamus, bilateral fusiform gyri, parahippocampal gyri, caudate nuclei, precuneus, supplementary motor area, temporal pole of middle temporal gyri, middle temporal gyri, inferior temporal gyri and midbrain (Table 2; Figure 1). Relative to HC group, ITE group demonstrated decreased FCS in right temporal pole of the superior temporal gyrus, middle temporal gyrus, temporal pole of the middle temporal gyrus and bilateral precuneus and increased FCS values in left triangular part of the inferior frontal gyrus, parahippocampal gyrus, supplementary motor area, caudate nucleus, right calcarine fissure and surrounding cortex, and midbrain (Table 3; Figure 2). As apposed to nITE group, ITE group found decreased FCS in the right medial superior frontal gyrus, left inferior temporal gyrus and bilateral fusiform gyri and increased FCS in right middle temporal gyrus, inferior temporal gyrus, middle occipital gyrus, and calcarine fissure and surrounding cortex (Table 4; Figure 3). In comparison to HC group, nITE group exhibited decreased FCS values in the bilateral middle temporal gyri, right orbital superior frontal gyrus, supplementary motor area, precuneus and superior frontal gyrus and increased FCS values in bilateral fusiform gyri, parahippocampal gyri and caudate nuclei, left temporal pole of the middle temporal gyrus, thalamus, right triangular part of the inferior frontal gyrus, and middle frontal gyrus (Table 5; Figure 4). Correlation analysis indicated that FCS values in the left caudate nucleus in children with ITE positively correlated with the VIQ (Figures 5A, B), while negatively correlated with disease duration (Figure 5C). FCS values in left middle temporal gyrus of nITE group demonstrated a positive correlation with VIQ, PIQ, and FIQ (Figures 6A–C). Besides, FCS in left caudate nucleus of nITE group were negatively correlated with VIQ, PIQ, and FIQ (Figures 6D–F). FCS values of abnormal brain regions in children with nITE and the disease course had no significant correlation.

**Table 2:** ANOVA of resting-state FCS was significantly different among the three groups.

**Brain regions**	**BA**	**Peak MNI coordinate**			***F*-value**	**Voxel value**
		***x***	***y***	***z***		
Left fusiform gyrus	20	−30	6	−48	12.23	68
Right fusiform gyrus	20	24	0	−36	17.11	81
Left parahippocampal gyrus	30	−24	−27	−18	7.40	62
Right parahippocampal gyrus	36	40	−21	−15	4.42	49
Left precuneus	7	−8	−70	52	5.30	58
Right precuneus	7	9	−66	42	6.05	81
Left caudate nucleus	-	−9	0	18	11.95	78
Right caudate nucleus	-	16	2	20	5.18	44
Left supplementary motor area	6	−6	6	60	4.99	47
Right supplementary motor area	6	12	−19	74	3.55	9
Right temporal pole of superior temporal gyrus	38	54	7	−15	7.42	39
Left temporal pole of middle temporal gyrus	35	−22	1	−34	4.37	26
Right temporal pole of middle temporal gyrus	21	57	3	−15	8.85	39
Left middle temporal gyrus	20	−51	−39	−9	5.59	79
Right middle temporal gyrus	21	42	−21	−15	6.26	59
Left inferior temporal gyrus	36	−30	6	−48	12.22	70
Right inferior temporal gyrus	20	37	0	−44	6.63	51
Right superior frontal gyrus	6	48	−24	9	4.43	20
Right medial superior frontal gyrus	9	6	51	48	6.38	40
Right orbital superior frontal gyrus	6	36	6	45	4.52	21
Right middle frontal gyrus	6	36	6	45	4.52	21
Left triangular inferior frontal gyrus	45	−48	24	18	7.53	97
Right triangular inferior frontal gyrus	45	48	30	18	6.88	76
Right precentral gyrus	4	15	−24	78	5.14	52
Right postcentral gyrus	1	51	−30	60	6.15	34
Left thalamus	-	−12	−8	17	8.10	58
Right calcarine fissure	18	21	−72	15	6.01	33
Right middle occipital gyrus	19	42	−81	6	4.79	21
Midbrain	-	0	−36	−18	4.58	17

BA, Brodmann area; MNI, Montreal Neurological Institute.

**Table 3:** Regions with altered FCS values in the intractable epilepsy group compared with the control group.

**Brain regions**	**BA**	**Peak MNI coordinate**			**Maximal *T*-value**	**Voxel value**
		***x***	***y***	***z***		
Left parahippocampal gyrus	30	−21	−27	−21	3.29	46
Left caudate nucleus	-	−9	−3	18	3.79	39
Left supplementary motor area	6	−6	6	60	2.76	43
Right precentral gyrus	4	15	−21	75	−2.86	25
Right postcentral gyrus	1	51	−30	60	2.98	28
Left triangular inferior frontal gyrus	48	−37	21	28	2.81	18
Right temporal pole of superior temporal gyrus	38	53	8	−13	−2.19	17
Right middle temporal gyrus	21	59	0	−16	−2.72	18
Right temporal pole of middle temporal gyrus	21	59	6	−17	−2.23	21
Left precuneus	7	−6	−69	51	−2.39	18
Right precuneus	7	9	−66	42	−3.02	44
Right calcarine fissure	18	21	−69	15	2.77	28
Midbrain	-	0	−36	−18	2.80	29

BA, Brodmann area; MNI, Montreal Neurological Institute.

*p* < 0.05, with Bonferroni correction.

**Table 4:** Regions with altered FCS values in the intractable epilepsy group compared with the non-intractable epilepsy group.

**Brain regions**	**BA**	**Peak MNI coordinate**			**Maximal *T*-value**	**Voxel value**
		***x***	***y***	***z***		
Right medial superior frontal gyrus	9	6	51	48	−3.00	29
Left inferior temporal gyrus	36	−32	1	−36	−2.09	20
Left fusiform gyrus	20	−30	3	−48	−3.49	25
Right fusiform gyrus	20	36	3	−48	−2.95	27
Right middle temporal gyrus	21	42	−21	−15	2.96	29
Right inferior temporal gyrus	20	57	−24	−27	2.79	27
Right middle occipital gyrus	19	42	−81	6	2.73	14
Right calcarine fissure	18	21	−72	15	2.26	15

BA, Brodmann area; MNI, Montreal Neurological Institute.

*p* < 0.05, with Bonferroni correction.

**Table 5:** Regions with altered FCS values in the non-intractable epilepsy group compared with the control group.

**Brain regions**	**BA**	**Peak MNI coordinate**			**Maximal *T*-value**	**Voxel value**
		***x***	***y***	***z***		
Left middle temporal gyrus	20	−51	−39	−9	−2.78	57
Right middle temporal gyrus	21	15	−36	−3	−3.13	88
Right orbital superior frontal gyrus	10	6	48	−9	−2.65	32
Right supplementary motor area	6	10	−15	78	−2.33	24
Right superior frontal gyrus	6	12	−15	78	−3.11	44
Right precuneus	7	3	−57	51	−2.71	82
Left fusiform gyrus	20	−27	−3	−39	4.08	91
Right fusiform gyrus	20	24	0	−3	4.88	63
Left parahippocampal gyrus	30	−24	−26	−17	2.81	66
Right parahippocampal gyrus	36	29	−14	−28	2.05	63
Left caudate nucleus	-	−9	0	15	3.72	88
Right caudate nucleus	-	12	8	21	2.76	35
Left temporal pole of middle temporal gyrus	35	−22	1	−34	2.18	32
Left thalamus	-	−9	−13	16	2.63	49
Right triangular inferior frontal gyrus	45	48	30	21	2.86	29
Right middle frontal gyrus	45	48	36	21	2.42	40

BA, Brodmann area; MNI, Montreal Neurological Institute.

*p* < 0.05, with Bonferroni correction.

## 4 Discussion

To our knowledge, it was the first study to investigate ITE in children using FCS analysis. The abnormal FCS of brain regions linked to ITE were primarily in the DMN and the prefrontal-limbic system. In comparison to nITE group, FCS abnormalities in ITE group were mainly concentrated in the temporal cortices and prefrontal cortices. Relative to HC group, nITE group demonstrated altered FCS in frontal lobe, temporal lobe and parietal lobe.

In our study, as we expected, the bilateral precuneus, right middle temporal gyrus, left triangular part of the inferior frontal gyrus, parahippocampal gyrus and supplementary motor area belong to the DMN (Koshino et al., 2014). The precuneus, as one of the key nodes of the DMN, is related to episodic memory, information processing and consciousness. The precuneus is also extensively connected to cortical and subcortical structures (Zhang and Li, 2012). Consistent with our finding of decreased FCS in the bilateral precuneus, Haneef et al. (2014) declared that decreased functional connectivity in the precuneus of ITE patients may be the pathological basis of memory impairment in temporal lobe epilepsy patients. The DMN associated with advanced cognitive functioning, for instance, learning, consciousness and episodic memory. Lopes et al. (2014) found that cognitive decline in patients with epilepsy was caused by frequent seizures, which might be related to inhibition of the DMN's function. Kay et al. (2013) found that DMN functional connectivity in idiopathic generalized epilepsy (IGE) patients was reduced and significantly reduced in those with refractory IGE and that DMN connectivity negatively correlated with disease course. Therefore, it has been speculated that DMN connectivity might be a biomarker of treatment resistance (Luo et al., 2011; McGill et al., 2012). We speculate that abnormal FCS in the above brain regions may be the cause of cognitive dysfunction, such as disruptions in memory and consciousness, in ITE patients.

In our study, changes in FCS values were found in left triangular part of the inferior frontal gyrus, supplementary motor area, parahippocampal gyrus, right temporal pole of the superior temporal gyrus and right temporal pole of the middle temporal gyrus that involved the prefrontal-limbic system. The prefrontal-limbic system includes prefrontal cortex, parahippocampal gyrus, amygdala, hippocampus and neocortex, including the temporal pole. The prefrontal-limbic system is a brain pathway regulating emotion and epilepsy (Braun, 2011; Chen et al., 2012). The limbic system may be regulated by the medial prefrontal cortex (Foland et al., 2008).

Prior studies have shown that the frontal lobe, temporal lobe and limbic system are all related to the pathogenesis of depression in epilepsy patients, and the hippocampus associated with anxiety disorders. The incidence of depression and anxiety disorders in epilepsy patients is higher than that in people without epilepsy. Thirty percent of patients with ITE suffer from depression or anxiety disorders, and most of them have intractable temporal lobe epilepsy (Kwon and Park, 2014). Prior studies confirmed that depression and anxiety are risk factors for ITE in epilepsy patients (Hitiris et al., 2007; Petrovski et al., 2010). Childhood epilepsy can be accompanied by anxiety, autism, depression and attention deficit hyperactivity disorder (ADHD) (Saute et al., 2014). In this study, we observed altered FCS with ITE were mainly observed in the prefrontal-limbic system, which may cause depression or anxiety in children with ITE.

Additionally, the temporal pole is an important site of seizures, and abnormalities in the temporal pole also contribute to lateralization of the epileptogenic focus (Chabardès et al., 2005). Kojan et al. (2018) found that the temporal pole showed hypometabolism using fluorodeoxyglucose positron emission tomography to study patients with mesial temporal lobe epilepsy (mTLE). Consistent with Kojan's studies, a decrease in FCS in right temporal pole of superior temporal gyrus and middle temporal gyrus was discovered in this study. We speculate that reduced FCS in the temporal pole may be closely related to recurrent seizures. In addition, the frontal lobe is closely correlated to consciousness, memory and executive functioning. Some research indicated frontal lobe function is abnormal, which leads to impaired working memory (Violante et al., 2017; Alagapan et al., 2019) and executive function (Pillai et al., 2017) and loss of consciousness (Bonini et al., 2016) in patients with epilepsy. Epilepsy is characterized as a network disorder, with each type of seizure involving unique cortical and subcortical networks that play a role in regulating and spreading ictal activity. One study of frontal lobe epilepsy (Rahatli et al., 2020) reported that cortical thickness in frontal lobe and extrafrontal lobes decreased, and the volume in multiple brain regions decreased. These findings may be related to epileptogenic foci in the frontal lobe and extrafrontal lobes, leading to the rapid spread of epileptic activity and the development of secondary epileptogenic foci. The frontal lobe contains extensive cortical and subcortical connections that can lead to the rapid spread of extrafrontal epileptic activity (including in the same hemisphere and the contralateral hemisphere), therefore making it difficult to localize epilepsy (Widjaja et al., 2011, 2014). Approximately 25% of refractory focal epilepsy cases are frontal lobe epilepsy. Abnormal neuronal connections are considered to be a key factor in seizures (Tailby et al., 2018). Decreased FCS in right medial superior frontal gyrus were discovered in ITE patients. Combined with previous studies, it is speculated that the recurrence of ITE after drug treatment may be related to the rapid spread of epileptic activity caused by extensive cortical and subcortical connections in the frontal lobe.

Relative to HC group, the nITE group presented altered FCS in frontal lobe, temporal lobe and parietal lobe. Zhong et al. (2018) reported that juvenile myoclonic epilepsy showed areas with reduced gray matter volume (GMV) predominantly in portions of the cerebral cortex, comprising the frontal area, temporal lobe and parieto-occipital lobe, which were linked with clinical presentation. The fusiform gyrus has been associated with face recognition and secondary classification of objects. It has the function of visual processing, and hallucinations occur when with increases in activity. On the account of the results of this study, we believe that increased FCS values in bilateral fusiform gyri in nITE group may be the cause of hallucinations and delusions. The parahippocampal gyrus and caudate nucleus are related to memory function. Thus, we speculate that increases in FCS in the bilateral parahippocampal gyri and caudate nuclei may be compensatory mechanisms in response to memory impairment in the nITE group.

Correlation analysis showed a correlation between FCS of left caudate nucleus and VIQ in both ITE and nITE groups, consistent with the previous literature (Wright et al., 2020). VIQ scores reflect cognitive functions such as speech comprehension, speech expression and auditory short-term memory. The caudate nucleus plays a crucial role in executive action, verbal fluency and language learning (Bick et al., 2019; Driscoll et al., 2024). The dorsal-posterior caudate is strongly associated with dorsolateral prefrontal cortex, whereas the ventral caudate primarily demonstrates interconnections with ventrolateral prefrontal cortex (Yeterian and Pandya, 1991; Leh et al., 2007). The impairment of prefrontal-caudate circuit may contribute to a decline in top-down control in the prefrontal cortex and compensated mechanisms may be induced in the caudate (Radulescu et al., 2013; Vink et al., 2014). Furthermore, increased brain activity in one cognitive control network was regarded as compensation for reduced activity in another network (Horowitz-Kraus et al., 2018). In Parkinson's disease, an increase in resting-state connectivity in the caudate, has previously been connected with cognitive maintenance (Manza et al., 2016). And the right caudate's increased network hubness appears to be a successful compensatory mechanism, which helps Parkinson's disease patients with normal cognitive function maintain their cognitive performance (Wright et al., 2020). De Simoni et al. (2018) confirmed that the decrease in functional connections between the caudate and anterior cingulate cortex in traumatic brain injury patients was related to executive and cognitive impairment. Our finding confirmed that the caudate nucleus was primarily associated with cognitive and executive functioning, and we speculate that the abnormal increase in FCS in the caudate nucleus may be a compensatory mechanism in response to cognitive impairment in epilepsy patients. A study in ITE patients found that caudate stimulation showed a significant improvement in learning, and confirmed that caudate was a promising neuromodulation target for improving memory (Bick et al., 2019). Moreover, Li et al. (2009) found that the caudate played a role in interictal spike-wave discharges and generalized seizures. In patients and animal models with ITE indicated that low-frequency stimulation of caudate nucleus in patients with ITE could inhibit interictal epileptic activity, and even the spread and generalization of seizures (Wagner et al., 1975; Chkhenkeli and Chkhenkeli, 1997; Chkhenkeli et al., 2004). Despite children with ITE being able to achieve significant seizure freedom through deep brain stimulation, it was not provided routinely, as it was still relatively new in pediatric populations (Yan et al., 2018). So, the caudate nucleus may become a key target for improving cognitive impairment and seizure frequency in children with ITE, providing more information for the clinical treatment of ITE. Our study support this as well. Besides, FCS in the caudate nucleus negatively correlated with disease duration. Kay et al. (2013) found that ITE patients showed significantly reduced DMN connectivity, which negatively correlated with epilepsy duration. Thus, we speculated that FCS changes in the left caudate nucleus might indicate the progression of ITE disease, showing the chronic damage to the brain functional network as a result of children with ITE. Also, FCS in left middle temporal gyrus of nITE patients positively correlated with VIQ, PIQ and FIQ. The middle temporal gyrus is reckoned to be an integration hub for semantic and phonological functions, which is crucial for sentence comprehension (Price, 2010). This study found reduced FCS in left middle temporal gyrus of the nITE group, which indicates that cognitive impairment may be linked to dysfunction in this brain region.

There are still some limitations. First, the study had a small sample size, there was no subtype grouping of patients with ITE, and our study reflected global changes in whole-brain functional connectivity strength. In addition, there may be a certain statistical bias. It is necessary to further this study to determine whether different types of seizures have different influence on FCS. Our study is initial, and we will keep on enlarging the database to address this problem. In addition, the HC group lacked a neuropsychological assessment, although we believe their neuropsychological status was good.

## 5 Conclusion

Our results discovered functional alterations in both the DMN and prefrontal-limbic system in children with ITE. The FCS values changes in the temporal cortices and prefrontal cortices may be related to drug resistance in children with ITE and contribute to early clinical diagnosis and preoperative localization. FCS can reveal intrinsic brain network dysfunction, which may contribute to our understanding of the neurophysiological and compensatory processes underlying ITE. FCS values in the prefrontal-limbic system are linked to cognitive impairment and affective disorders in ITE patients. And our findings also suggest that the caudate may become a key target for improving cognitive impairment and seizures in children with ITE, providing more information for the clinical treatment of ITE.

## Data availability statement

The original contributions presented in the study are included in the article/supplementary material, further inquiries can be directed to the corresponding authors.

## Ethics statement

The studies involving humans were approved by the Ethics Committee of the Affiliated Hospital of Zunyi Medical University. The studies were conducted in accordance with the local legislation and institutional requirements. Written informed consent for participation in this study was provided by the participants' legal guardians/next of kin.

## Author contributions

XL: Conceptualization, Data curation, Investigation, Methodology, Software, Visualization, Writing—original draft. HL: Supervision, Validation, Writing—review & editing. TZ: Supervision, Validation, Writing—review & editing.

## References

[B1] Aaberg K. M. Gunnes N. Bakken I. J. Lund Søraas C. Berntsen A. Magnus P.. (2017). Incidence and prevalence of childhood epilepsy: a nationwide cohort study. Pediatrics 139, 3908. 10.1542/peds.2016-3908 28557750

[B2] Alagapan S. Lustenberger C. Hadar E. Shin H. W. Fröhlich F. (2019). Low-frequency direct cortical stimulation of left superior frontal gyrus enhances working memory performance. Neuroimage 184, 697–706. 10.1016/j.neuroimage.2018.09.064 30268847 PMC6240347

[B3] Anyanwu C. Motamedi G. K. (2018). Diagnosis and surgical treatment of drug-resistant epilepsy. Brain Sci. 8, 49. 10.3390/brainsci8040049 29561756 PMC5924385

[B4] Bick S. K. Patel S. R. Katnani H. A. Peled N. Widge A. Cash S. S.. (2019). Caudate stimulation enhances learning. Brain 142, 2930–2937. 10.1093/brain/awz254 31504220

[B5] Boerwinkle V. L. Mohanty D. Foldes S. T. Guffey D. Minard C. G. Vedantam A.. (2017). Correlating resting-state functional magnetic resonance imaging connectivity by independent component analysis-based epileptogenic zones with intracranial electroencephalogram localized seizure onset zones and surgical outcomes in prospective pediatric intractable epilepsy study. Brain Connect 7, 424–442. 10.1089/brain.2016.0479 28782373 PMC5647510

[B6] Bonini F. Lambert I. Wendling F. McGonigal A. Bartolomei F. (2016). Altered synchrony and loss of consciousness during frontal lobe seizures. Clin. Neurophysiol. 127, 1170–1175. 10.1016/j.clinph.2015.04.050 25912335

[B7] Braun K. (2011). The prefrontal-limbic system: development, neuroanatomy, function, and implications for socioemotional development. Clin. Perinatol. 38, 685–702. 10.1016/j.clp.2011.08.013 22107898

[B8] Chabardès S. Kahane P. Minotti L. Tassi L. Grand S. Hoffmann D.. (2005). The temporopolar cortex plays a pivotal role in temporal lobe seizures. Brain 128, 1818–1831. 10.1093/brain/awh512 15857932

[B9] Chen S. Wu X. Lui S. Wu Q. Yao Z. Li Q.. (2012). Resting-state fMRI study of treatment-naïve temporal lobe epilepsy patients with depressive symptoms. Neuroimage 60, 299–304. 10.1016/j.neuroimage.2011.11.092 22178816

[B10] Chkhenkeli S. A. Chkhenkeli I. S. (1997). Effects of therapeutic stimulation of nucleus caudatus on epileptic electrical activity of brain in patients with intractable epilepsy. Stereotact. Funct. Neurosurg. 69, 221–224. 10.1159/000099878 9711758

[B11] Chkhenkeli S. A. Sramka M. Lortkipanidze G. S. Rakviashvili T. N. Bregvadze E. Magalashvili G. E.. (2004). Electrophysiological effects and clinical results of direct brain stimulation for intractable epilepsy. Clin. Neurol. Neurosurg. 106, 318–329. 10.1016/j.clineuro.2004.01.009 15297008

[B12] De Simoni S. Jenkins P. O. Bourke N. J. Fleminger J. J. Hellyer P. J. Jolly A. E.. (2018). Altered caudate connectivity is associated with executive dysfunction after traumatic brain injury. Brain 141, 148–164. 10.1093/brain/awx309 29186356 PMC5837394

[B13] Diao Q. Liu J. Zhang X. (2020). Enhanced positive functional connectivity strength in left-sided chronic subcortical stroke. Brain Res. 1733, 146727. 10.1016/j.brainres.2020.146727 32061738

[B14] Dlouhy B. J. Gehlbach B. K. Richerson G. B. (2016). Sudden unexpected death in epilepsy: basic mechanisms and clinical implications for prevention. J. Neurol. Neurosurg. Psychiatry 87, 402–413. 10.1136/jnnp-2013-307442 26979537

[B15] Dong C. Liu T. Wen W. Kochan N. A. Jiang J. Li Q.. (2018). Altered functional connectivity strength in informant-reported subjective cognitive decline: a resting-state functional magnetic resonance imaging study. Alzheimers Dement 10, 688–697. 10.1016/j.dadm.2018.08.011 30426065 PMC6222034

[B16] Driscoll M. E. Bollu P. C. Tadi P. (2024). Neuroanatomy, Nucleus Caudate StatPearls. Treasure Island, FL: Ineligible Companies. Disclosure: Pradeep Bollu declares no relevant financial relationships with ineligible companies. Disclosure: Prasanna Tadi declares no relevant financial relationships with ineligible companies: StatPearls Publishing Copyright© 2024, StatPearls Publishing LLC.

[B17] Falco-Walter J. (2020). Epilepsy-definition, classification, pathophysiology, and epidemiology. Semin. Neurol. 40, 617–623. 10.1055/s-0040-1718719 33155183

[B18] Foland L. C. Altshuler L. L. Bookheimer S. Y. Eisenberger N. Townsend J. Thompson P. M. (2008). Evidence for deficient modulation of amygdala response by prefrontal cortex in bipolar mania. Psychiatry Res. 162, 27–37. 10.1016/j.pscychresns.2007.04.007 18063349 PMC2410029

[B19] Haneef Z. Lenartowicz A. Yeh H. J. Levin H. S. Engel J. Jr. Stern J. M. (2014). Functional connectivity of hippocampal networks in temporal lobe epilepsy. Epilepsia 55, 137–145. 10.1111/epi.12476 24313597 PMC3946924

[B20] Hitiris N. Mohanraj R. Norrie J. Sills G. J. Brodie M. J. (2007). Predictors of pharmacoresistant epilepsy. Epilepsy Res. 75, 192–196. 10.1016/j.eplepsyres.2007.06.003 17628429

[B21] Horowitz-Kraus T. Woodburn M. Rajagopal A. Versace A. L. Kowatch R. A. Bertocci M. A.. (2018). Decreased functional connectivity in the fronto-parietal network in children with mood disorders compared to children with dyslexia during rest: an fMRI study. Neuroimage Clin. 18, 582–590. 10.1016/j.nicl.2018.02.034 29845006 PMC5964829

[B22] Hu J. Ran H. Chen G. He Y. Li Q. Liu J.. (2023). Altered neurovascular coupling in children with idiopathic generalized epilepsy. CNS Neurosci. Ther. 29, 609–618. 10.1111/cns.14039 36480481 PMC9873522

[B23] Ibrahim G. M. Morgan B. R. Lee W. Smith M. L. Donner E. J. Wang F.. (2014). Impaired development of intrinsic connectivity networks in children with medically intractable localization-related epilepsy. Hum. Brain Mapp. 35, 5686–5700. 10.1002/hbm.22580 24976288 PMC6869397

[B24] Jiang L. W. Qian R. B. Fu X. M. Zhang D. Peng N. Niu C. S.. (2018). Altered attention networks and DMN in refractory epilepsy: a resting-state functional and causal connectivity study. Epilepsy Behav. 88, 81–86. 10.1016/j.yebeh.2018.06.045 30243110

[B25] Kay B. P. DiFrancesco M. W. Privitera M. D. Gotman J. Holland S. K. Szaflarski J. P. (2013). Reduced default mode network connectivity in treatment-resistant idiopathic generalized epilepsy. Epilepsia 54, 461–470. 10.1111/epi.12057 23293853 PMC3593969

[B26] Kojan M. DoleŽalová I. Koritáková E. Mareček R. Rehák Z. Hermanová M.. (2018). Predictive value of preoperative statistical parametric mapping of regional glucose metabolism in mesial temporal lobe epilepsy with hippocampal sclerosis. Epilepsy Behav. 79, 46–52. 10.1016/j.yebeh.2017.11.014 29247965

[B27] Koshino H. Minamoto T. Yaoi K. Osaka M. Osaka N. (2014). Coactivation of the default mode network regions and working memory network regions during task preparation. Sci. Rep. 4, 5954. 10.1038/srep05954 25092432 PMC4121601

[B28] Kwan P. Arzimanoglou A. Berg A. T. Brodie M. J. Allen Hauser W. Mathern G.. (2010). Definition of drug resistant epilepsy: consensus proposal by the *ad hoc* Task Force of the ILAE commission on therapeutic strategies. Epilepsia 51, 1069–1077. 10.1111/j.1528-1167.2009.02397.x 19889013

[B29] Kwon O. Y. Park S. P. (2014). Depression and anxiety in people with epilepsy. J. Clin. Neurol. 10, 175–188. 10.3988/jcn.2014.10.3.175 25045369 PMC4101093

[B30] Leh S. E. Ptito A. Chakravarty M. M. Strafella A. P. (2007). Fronto-striatal connections in the human brain: a probabilistic diffusion tractography study. Neurosci. Lett. 419, 113–118. 10.1016/j.neulet.2007.04.049 17485168 PMC5114128

[B31] Li Q. Luo C. Yang T. Yao Z. He L. Liu L.. (2009). EEG-fMRI study on the interictal and ictal generalized spike-wave discharges in patients with childhood absence epilepsy. Epilepsy Res. 87, 160–168. 10.1016/j.eplepsyres.2009.08.018 19836209

[B32] Li Y. Tan Z. Wang J. Wang Y. Gan Y. Wen F.. (2017). Alterations in spontaneous brain activity and functional network reorganization following surgery in children with medically refractory epilepsy: a resting-state functional magnetic resonance imaging study. Front. Neurol. 8, 374. 10.3389/fneur.2017.00374 28824531 PMC5541057

[B33] Li Y. Wang X. Li Y. Sun Y. Sheng C. Li H.. (2016). Abnormal resting-state functional connectivity strength in mild cognitive impairment and its conversion to Alzheimer's disease. Neural Plast. 2016, 4680972. 10.1155/2016/4680972 26843991 PMC4710946

[B34] Lopes R. Moeller F. Besson P. Ogez F. Szurhaj W. Leclerc X.. (2014). Study on the relationships between intrinsic functional connectivity of the default mode network and transient epileptic activity. Front. Neurol. 5, 201. 10.3389/fneur.2014.00201 25346721 PMC4193009

[B35] Luo C. Li Q. Lai Y. Xia Y. Qin Y. Liao W.. (2011). Altered functional connectivity in default mode network in absence epilepsy: a resting-state fMRI study. Hum. Brain Mapp. 32, 438–449. 10.1002/hbm.21034 21319269 PMC6870112

[B36] Manza P. Zhang S. Li C. S. Leung H. C. (2016). Resting-state functional connectivity of the striatum in early-stage Parkinson's disease: cognitive decline and motor symptomatology. Hum. Brain Mapp. 37, 648–662. 10.1002/hbm.23056 26566885 PMC4843498

[B37] McGill M. L. Devinsky O. Kelly C. Milham M. Castellanos F. X. Quinn B. T.. (2012). Default mode network abnormalities in idiopathic generalized epilepsy. Epilepsy Behav. 23, 353–359. 10.1016/j.yebeh.2012.01.013 22381387 PMC4407647

[B38] Petrovski S. Szoeke C. E. Jones N. C. Salzberg M. R. Sheffield L. J. Huggins R. M.. (2010). Neuropsychiatric symptomatology predicts seizure recurrence in newly treated patients. Neurology 75, 1015–1021. 10.1212/WNL.0b013e3181f25b16 20837970

[B39] Pillai S. H. Raghavan S. Mathew M. Gopalan G. M. Kesavadas C. Sarma S.. (2017). Juvenile myoclonic epilepsy with frontal executive dysfunction is associated with reduced gray matter volume by voxel-based morphometry. Ann. Indian Acad. Neurol. 20, 270–273. 10.4103/aian.AIAN_44_17 28904460 PMC5586123

[B40] Pressl C. Brandner P. Schaffelhofer S. Blackmon K. Dugan P. Holmes M.. (2019). Resting state functional connectivity patterns associated with pharmacological treatment resistance in temporal lobe epilepsy. Epilepsy Res. 149, 37–43. 10.1016/j.eplepsyres.2018.11.002 30472489 PMC6483378

[B41] Price C. J. (2010). The anatomy of language: a review of 100 fMRI studies published in 2009. Ann. N. Y. Acad. Sci. 1191, 62–88. 10.1111/j.1749-6632.2010.05444.x 20392276

[B42] Radulescu E. Minati L. Ganeshan B. Harrison N. A. Gray M. A. Beacher F. D.. (2013). Abnormalities in fronto-striatal connectivity within language networks relate to differences in grey-matter heterogeneity in Asperger syndrome. Neuroimage Clin. 2, 716–726. 10.1016/j.nicl.2013.05.010 24179823 PMC3777793

[B43] Rahatli F. K. Sezer T. Has A. C. Agildere A. M. (2020). Evaluation of cortical thickness and brain volume on 3 Tesla magnetic resonance imaging in children with frontal lobe epilepsy. Neurol. Sci. 41, 825–833. 10.1007/s10072-019-04135-4 31802343

[B44] Saute R. Dabbs K. Jones J. E. Jackson D. C. Seidenberg M. Hermann B. P. (2014). Brain morphology in children with epilepsy and ADHD. PLoS ONE 9, e95269. 10.1371/journal.pone.0095269 24760032 PMC3997349

[B45] Shi Y. Li J. Feng Z. Xie H. Duan J. Chen F.. (2020). Abnormal functional connectivity strength in first-episode, drug-naïve adult patients with major depressive disorder. Prog Neuropsychopharmacol. Biol. Psychiatry 97, 109759. 10.1016/j.pnpbp.2019.109759 31499128

[B46] Songjiang L. Tijiang Z. Heng L. Wenjing Z. Bo T. Ganjun S.. (2021). Impact of brain functional network properties on intelligence in children and adolescents with focal epilepsy: a resting-state MRI study. Acad. Radiol. 28, 225–232. 10.1016/j.acra.2020.01.004 32037257

[B47] Tailby C. Kowalczyk M. A. Jackson G. D. (2018). Cognitive impairment in epilepsy: the role of reduced network flexibility. Ann. Clin. Transl. Neurol. 5, 29–40. 10.1002/acn3.503 29376090 PMC5771327

[B48] Tang F. Hartz A. M. S. Bauer B. (2017). Drug-resistant epilepsy: multiple hypotheses, few answers. Front. Neurol. 8, 301. 10.3389/fneur.2017.00301 28729850 PMC5498483

[B49] Vannest J. Radhakrishnan R. Gutierrez-Colina A. M. Wade S. L. Maloney T. Combs A.. (2021). Altered functional network connectivity and working memory dysfunction in adolescents with epilepsy. Brain Imaging Behav. 15, 2513–2523. 10.1007/s11682-021-00452-5 33528802

[B50] Vink M. Zandbelt B. B. Gladwin T. Hillegers M. Hoogendam J. M. van den Wildenberg W. P.. (2014). Frontostriatal activity and connectivity increase during proactive inhibition across adolescence and early adulthood. Hum. Brain Mapp. 35, 4415–4427. 10.1002/hbm.22483 24532023 PMC6869143

[B51] Violante I. R. Li L. M. Carmichael D. W. Lorenz R. Leech R. Hampshire A.. (2017). Externally induced frontoparietal synchronization modulates network dynamics and enhances working memory performance. Elife 6, 20. 10.7554/eLife.22001.020 28288700 PMC5349849

[B52] Wagner R. II Feeney D. M. Gullotta F. P. Cote I. L. (1975). Suppression of cortical epileptiform activity by generalized and localized ECoG desynchronization. Electroencephalogr. Clin. Neurophysiol. 39, 499–506. 10.1016/0013-4694(75)90051-6 52443

[B53] Wang L. Xia M. Li K. Zeng Y. Su Y. Dai W.. (2015). The effects of antidepressant treatment on resting-state functional brain networks in patients with major depressive disorder. Hum. Brain Mapp. 36, 768–778. 10.1002/hbm.22663 25332057 PMC6869500

[B54] Wang S. Zhan Y. Zhang Y. Lv L. Wu R. Zhao J.. (2017). Abnormal functional connectivity strength in patients with adolescent-onset schizophrenia: a resting-state fMRI study. Eur. Child Adolesc. Psychiatry 26, 839–845. 10.1007/s00787-017-0958-2 28185094

[B55] Wang Z. Wang X. Rong R. Xu Y. Zhang B. Wang Z. (2019). Impaired hippocampal functional connectivity in patients with drug resistant, generalized tonic-clonic seizures. Neuroreport 30, 700–706. 10.1097/WNR.0000000000001262 31116131 PMC6571184

[B56] Wechsler D. (1949). Wechsler Intelligence Scale for Children; Manual. Oxford, England: The Psychological Corp.

[B57] Widjaja E. Kis A. Go C. Snead O. C. III Smith M. L. (2014). Bilateral white matter abnormality in children with frontal lobe epilepsy. Epilepsy Res. 108, 289–294. 10.1016/j.eplepsyres.2013.12.001 24380759 PMC3951988

[B58] Widjaja E. Mahmoodabadi S. Z. Snead O. C. III Almehdar A. Smith M. L. (2011). Widespread cortical thinning in children with frontal lobe epilepsy. Epilepsia 52, 1685–1691. 10.1111/j.1528-1167.2011.03085.x 21627647

[B59] Wright N. Alhindi A. Millikin C. Modirrousta M. Udow S. Borys A.. (2020). Elevated caudate connectivity in cognitively normal Parkinson's disease patients. Sci. Rep. 10, 17978. 10.1038/s41598-020-75008-6 33087833 PMC7578639

[B60] Yan H. Toyota E. Anderson M. Abel T. J. Donner E. Kalia S. K.. (2018). A systematic review of deep brain stimulation for the treatment of drug-resistant epilepsy in childhood. J. Neurosurg. Pediatr. 23, 274–284. 10.3171/2018.9.PEDS18417 30544364

[B61] Yang F. Jia W. Kukun H. Ding S. Zhang H. Wang Y. (2022). A study of spontaneous brain activity on resting-state functional magnetic resonance imaging in adults with MRI-negative temporal lobe epilepsy. Neuropsychiatr. Dis. Treat. 18, 1107–1116. 10.2147/NDT.S366189 35677937 PMC9170234

[B62] Yeterian E. H. Pandya D. N. (1991). Prefrontostriatal connections in relation to cortical architectonic organization in rhesus monkeys. J. Comp. Neurol. 312, 43–67. 10.1002/cne.903120105 1744243

[B63] Yu B. Kong F. Peng M. Ma H. Liu N. Guo Q. (2012). Assessment of memory/attention impairment in children with primary nocturnal enuresis: a voxel-based morphometry study. Eur. J. Radiol. 81, 4119–4122. 10.1016/j.ejrad.2012.01.006 22939366

[B64] Zhang S. Li C. S. (2012). Functional connectivity mapping of the human precuneus by resting state fMRI. Neuroimage 59, 3548–3562. 10.1016/j.neuroimage.2011.11.023 22116037 PMC3288461

[B65] Zhong C. Liu R. Luo C. Jiang S. Dong L. Peng R.. (2018). Altered structural and functional connectivity of juvenile myoclonic epilepsy: an fMRI study. Neural. Plast. 2018, 7392187. 10.1155/2018/7392187 29681927 PMC5846383

[B66] Zhu J. Xu C. Zhang X. Qiao L. Wang X. Zhang X.. (2021). Altered amplitude of low-frequency fluctuations and regional homogeneity in drug-resistant epilepsy patients with vagal nerve stimulators under different current intensity. CNS Neurosci. Ther. 27, 320–329. 10.1111/cns.13449 32965801 PMC7871792

